# Supplementary material for: Platelet Mitochondrial Respiration, Endogenous Coenzyme Q10 and Oxidative Stress in Patients with Chronic Kidney Disease
Source: Diagnostics (Basel). 2020 Mar 23;10(3):176. doi: 10.3390/diagnostics10030176 (PMC7151406; doi:10.3390/diagnostics10030176)
Supplement: Supplementary file 1 [file diagnostics-10-00176-s001.pdf]

Platelet Mitochondrial Respiration, Endogenous Coenzyme Q_{10} and Oxidative Stress in Patients with Chronic Kidney Disease

Anna Gvozdjáková [1,*], Zuzana Sumbalová [1], Jarmila Kucharská [1], Mária Komlósi [2], Zuzana Rausová [1], Oľga Vančová [1], Monika Számošová [2] and Viliam Mojto [2]

1 Pharmacobiochemical Laboratory of 3rd Department of Internal Medicine, Faculty of Medicine, Comenius University in Bratislava, Sasinkova 4, 811 08 Bratislava, Slovakia; zuzana.sumbalova@fmed.uniba.sk (Z.S.); jarmila.kucharska@fmed.uniba.sk (J.K.); zuzana.rausova@fmed.uniba.sk (Z.R.); olga.vancova@fmed.uniba.sk (O.V)

2 Faculty of Medicine, 3rd Department of Internal Medicine, Comenius University in Bratislava, 4, 811 08 Bratislava, Slovakia; komlosi.mar@gmail.com (M.K.); monika.szamosova@gmail.com (M.S.); viliam.mojto@gmail.com (V.M.)

* Correspondence: anna.gvozdjakova@fmed.uniba.sk; Tel: +421-2-9011-9242

Received: 30 January 2020; Accepted: 19 March 2020; Published: 22 March 2020

Supplementary Material

Table 1. Metabolic parameters of human volunteers and groups of CKD patients.

**Table S1a.**	**Kidney parameters of human volunteers and groups of CKD patients**				
**Kidney**					
		**Control**	**CKD-ALL**	**CKD+AH**	**CKD+AH+DM**
**eGFR**	**mean**	**1.328**	**0.578**	**0.574**	**0.583**
(mL·s^{-1}·1.73m^{-2})	**stdev P**	0.239	0.220	0.211	0.234
	**sem**	**0.069**	**0.043**	**0.053**	**0.074**
	**n**	12	26	16	10
	P vs Control		**0.000000**	**0.000003**	**0.000001**
**Creatinine**	**mean**	**70.7**	**194.3**	**198.5**	**187.3**
(μmol.L^{-1})	**stdev P**	17.7	88.2	80.9	99.0
	**sem**	**5.10**	**16.98**	**19.62**	**31.32**
	**n**	12	27	17	10
	P vs Control		**0.00004**	**0.00002**	**0.0011**
**Uric acid**	**mean**	**287.4**	**370.8**	**346.8**	**409.2**
(μmol.L^{-1})	**stdev P**	45.5	127.7	103.5	151.4
	**sem**	**13.7**	**25.05**	**25.86**	**47.86**
	**n**	11	26	16	10
	P vs Control		**0.048**	0.10	**0.026**

**Table S1b.**	**Blood parameters of human volunteers and all CKD patients**				
**Blood**					
		**Control**	**CKD-ALL**	**CKD+AH**	**CKD+AH+DM**
**Hgb**	**mean**	**140.5**	**129.9**	**132.9**	**124.7**
(g.L^{-1})	**stdev P**	7.6	22.8	19.9	26.2
	**sem**	**2.2**	**4.38**	**4.82**	**8.28**

	**n**	12	27	17	10
	P vs Control		0.13	0.2	0.072
**CRP**	**mean**	**1.729**	**7.213**	**6.800**	**7.833**
($g.L^{-1}$)	**stdev P**	0.918	6.317	5.560	7.262
	**sem**	**0.277**	**1.263**	**1.436**	**2.296**
	**n**	11	25	15	10
	P vs Control		**0.0003**	**0.008**	**0.017**
**Glucose**	**mean**	**5.783**	**6.19**	**5.794**	**6.870**
($mmol.L^{-1}$)	**stdev P**	0.574	1.82	0.980	2.565
	**sem**	**0.166**	**0.35**	**0.238**	**0.811**
	**n**	12	27	17	10
	P vs Control		0.5	1.0	0.2

Table S1c. **Lipids parameters of human volunteers and all CKD patients**

**Lipids**		**Control**	**CKD-ALL**	**CKD+AH**	**CKD+AH+DM**
**TAG**	**mean**	**1.16**	**1.78**	**1.71**	**1.92**
($mmol.L^{-1}$)	**stdev P**	0.37	0.89	0.83	0.98
	**sem**	**0.12**	**0.18**	**0.21**	**0.35**
	**n**	9	24	16	8
	P vs Control		0.057	0.081	0.062
**LDL-Chol**	**mean**	**3.750**	**3.55**	**3.50**	**3.651**
($mmol.L^{-1}$)	**stdev P**	0.397	1.01	1.03	0.950
	**sem**	**0.132**	**0.21**	**0.26**	**0.336**
	**n**	9	24	16	8
	P vs Control		0.6	0.5	0.8
**HDL-Chol**	**mean**	**1.50**	**1.31**	**1.33**	**1.27**
($mmol.L^{-1}$)	**stdev P**	0.23	0.34	0.35	0.31
	**sem**	**0.07**	**0.07**	**0.09**	**0.10**
	**n**	10	25	16	9
	P vs Control		0.12	0.2	0.10
**Chol-Total**	**mean**	**5.70**	**5.55**	**5.30**	**6.00**
($mmol.L^{-1}$)	**stdev P**	0.45	1.54	1.40	1.67
	**sem**	**0.15**	**0.31**	**0.35**	**0.56**
	**n**	9	25	16	9
	P vs Control		0.8	0.4	0.6

Table S1d. **Liver parameters of human volunteers and all CKD patients**

**Liver**		**Control**	**CKD-ALL**	**CKD+AH**	**CKD+AH+DM**
**AST**		**0.371**	**0.405**	**0.393**	**0.421**
($kat.L^{-1}$)	**stdev P**	0.099	0.177	0.132	0.222
	**sem**	**0.028**	**0.037**	**0.037**	**0.070**

	**n**	12	23	13	10
	P vs Control		0.5	0.7	0.5
**ALP**	**mean**	**0.297**	**0.863**	**0.930**	**0.756**
(kat.L^{-1})	**stdev P**	0.060	0.757	0.812	0.645
	**sem**	**0.017**	**0.148**	**0.203**	**0.204**
	**n**	12	26	16	10
	P vs Control		**0.001**	**0.015**	**0.030**
**GMT**	**mean**	**0.751**	**0.530**	**0.505**	**0.566**
(kat.L^{-1})	**stdev P**	0.367	0.332	0.317	0.350
	**sem**	**0.130**	**0.066**	**0.082**	**0.111**
	**n**	8	25	15	10
	P vs Control		0.13	0.12	0.32

Table S2. Platelet mitochondrial function in control subjects and groups of CKD patients.

		O2 flux (pmol·s^{-1}·10^{-6} cells)			
		**Control**	**CKD-ALL**	**CKD+AH**	**CKD+AH+DM**
**ce1**	**mean**	0.245	0.230	0.233	0.246
	**stdev P**	0.067	0.058	0.064	0.041
	**sem**	0.019	0.011	0.016	0.014
	**n**	12	26	17	9
**1PM**	**mean**	0.052	0.045	0.042	0.056
	**stdev P**	0.038	0.022	0.019	0.022
	**sem**	0.011	0.004	0.005	0.007
	**n**	12	27	17	10
**2D**	**mean**	0.204	0.218	0.214	0.252
	**stdev P**	0.124	0.101	0.106	0.090
	**sem**	0.036	0.019	0.026	0.029
	**n**	12	27	17	10
**2c**	**mean**	0.202	0.227	0.225	0.257
	**stdev P**	0.115	0.101	0.107	0.086
	**sem**	0.033	0.020	0.027	0.027
	**n**	12	26	16	10
**3U**	**mean**	0.233	0.258	0.255	0.291
	**stdev P**	0.134	0.105	0.106	0.092
	**sem**	0.039	0.020	0.026	0.029
	**n**	12	27	17	10
**4G**	**mean**	0.284	0.302	0.299	0.339
	**stdev P**	0.157	0.135	0.142	0.118
	**sem**	0.047	0.026	0.034	0.037
	**n**	11	27	17	10
**5S**	**mean**	0.772	0.767	0.770	0.831
	**stdev P**	0.188	0.209	0.238	0.186
	**sem**	0.057	0.040	0.058	0.059
	**n**	11	27	17	10
**6Rot**	**mean**	0.545	0.541	0.539	0.586
	**stdev P**	0.108	0.133	0.140	0.150
	**sem**	0.031	0.026	0.034	0.048
	**n**	12	27	17	10

Table S3. Endogenous $CoQ_{10\text{-TOTAL}}$ concentration in platelets, blood and plasma in control subjects and groups of CKD patients.

$CoQ_{10\text{-TOTAL}}$					
		**Control**	**CKD-ALL**	**CKD+AH**	**CKD+AH+DM**
**Platelets**					
(pmol.10^{-9}cells)	**mean**	156.1	115.7	100.7	133.4
	**stdev P**	24.78	43.70	29.56	37.80
	**sem**	7.47	8.92	7.90	12.60
	**n**	11	24	14	9
	P vs Control		**0.009**	**0.00008**	0.14
**Blood**	**mean**	0.277	0.226	0.210	0.241
(µmol.L^{-1})	**stdev P**	0.131	0.06	0.066	0.013
	**sem**	0.038	0.013	0.018	0.005
	**n**	11	22	14	7
	P vs Control		0.14	0.12	0.51
**Plasma**	**mean**	0.398	0.278	0.268	0.285
(µmol.L^{-1})	**stdev P**	0.143	0.092	0.097	0.064
	**sem**	0.043	0.021	0.026	0.029
	**n**	11	20	14	5
	P vs Control		**0.010**	**0.016**	0.13

Table S4. Physical parameters of human volunteers and groups of CKD patients.

		**Control**	**CKD-ALL**	**CKD+AH**	**CKD+AH+DM**
**Age**	**mean**	**67.8**	**58.3**	**54.9**	**64.0**
(years)	**stdev P**	7.1	8.7	9.1	3.6
	**sem**	**2.0**	**1.68**	**2.20**	**1.13**
	**n**	12	27	17	10
	P vs Control		**0.002**	**0.0005**	0.15
**BMI**	**mean**	**23.9**	**27.4**	**26.9**	**28.3**
(kg.m^{-2})	**stdev P**	1.8	5.8	5.7	5.7
	**sem**	**0.6**	**1.11**	**1.39**	**1.79**
	**n**	10	27	17	10
	P vs Control		0.077	0.14	**0.040**
